# Primary Cough Headache Disorder Responds to Low Volume Therapeutic Lumbar Puncture: A Case Report With a Side Note on Therapeutics and Cranio-Spinal Dissociation

**DOI:** 10.7759/cureus.10262

**Published:** 2020-09-05

**Authors:** Hassan Kesserwani

**Affiliations:** 1 Neurology, Flowers Medical Group, Dothan, USA

**Keywords:** headache treatment, severe headache

## Abstract

Primary cough headache disorder (PCHD) is a unique disorder with an underlying dynamic cause. Having ruled out an underlying specific etiology, it is ipso facto a diagnosis of exclusion. It has been empirically treated with cerebrospinal fluid (CSF) pressure-lowering modalities; such as indomethacin, acetazolamide and high volume CSF drainage by lumbar puncture. We describe the case of a 66-year-old man with at least a 20-year history of PCHD, who dramatically responded to low volume CSF drainage, totaling three lumbar punctures over the course of twenty years, with rapid and effective relief of headache. We review the therapy of PCHD and discuss the CSF dynamics as it pertains to lumbar and cisterna magna CSF pressures. We also propose potential mechanisms for the effectiveness of CSF lowering measures.

## Introduction

Primary cough headache disorder (PCHD) is also referred to as the benign cough headache disorder or Valsalva-induced headache. The third edition of the International Classification of Headache Disorders (ICHD-3) classifies it as a primary cough headache triggered by coughing or a Valsalva maneuvre such as straining, sneezing, yelling, bending, stooping or weight-lifting; all events that transiently increase intracranial pressure. The headache is of sudden onset and is transient, lasting anywhere from one second to two hours [1]. PCHD usually occurs after the age of 40. The headache is usually bilateral and occipital but can be bifrontal. It is a diagnosis of exclusion and necessitates a normal magnetic resonance imaging (MRI) of the brain with no structural or other space-occupying lesions. A gadolinium-enhancing image may be necessary to rule out a lepto- or pachymeningitis. Cerebrospinal fluid (CSF) hypotension may manifest with leptomeningeal enhancement in 80 % of cases and may present with a cough headache. Funduscopic eye examination is necessary to rule out papilledema. It is also important to rule out basilar impression or platybasia and the cerebellar ectopia that is seen with the Arnold Chiari malformation, type I. The latter is a frequent cause of symptomatic cough headaches. Treatment strategies have included indomethacin, doses ranging from 50 to 200 milligrams (mg) per day, acetazolamide, and high volume, 40 cubic centimeter (cc) CSF drainage, with a therapeutic lumbar puncture [2-4]. These treatment modalities are postulated to lower the intracranial pressure.

Coughing and the other events associated with the Valsalva maneuvre listed above, result in increased intra-thoracic and intra-abdominal pressure. This impedes venous return to the right atrium. This increases central venous pressure (CVP) which subsequently increases intracranial pressure (ICP). The jugular venous system and epidural venous plexus being valveless, leads to the transmission of CVP into these channels. The surge of blood into the epidural venous plexus leads to compression of the dura, which leads to an increase in CSF subarachnoid pressure [3]. Hence CSF pressure lowering therapeutic modalities make intuitive sense.

In this case report, we describe a 66-year-old man with at least a 20-year history of PCHD; who responded dramatically to three low volume, three cc CSF, therapeutic lumbar punctures, over the course of 20 years. Indomethacin was not considered due to his history of gastroesophageal reflux disease. Acetazolamide dosing at one gram per day was ineffective and side effects were intolerable. There are no placebo-controlled studies to determine the effectiveness of these therapies and all experience is anecdotal. Neil Raskin had suggested high-volume, 40 cc, CSF drainage and there are no case reports of low volume CSF drainage [2]. We feel that the latter approach is associated with less morbidity; back pain and CSF hypotension headache. In this article, we discuss the potential pathophysiology of cough headaches and outline in detail the dynamics of lumbar and cisterna magna CSF pressures. We discuss the potential mechanisms of therapeutic CSF drainage and the mechanisms of pharmacotherapy in PCHD.

## Case presentation

We describe the case of a 66-year-old man who was periodically evaluated in the clinic over the course of 20 years. His primary complaint was an overwhelming bifrontal intense pressure-like headache lasting about thirty seconds brought on immediately by coughing, sneezing and straining with weights. The headache was not triggered by stooping or straining during bowel motion. It resolved relatively quickly after thirty seconds. He saw another neurologist who prescribed a beta-blocker, propranolol, without much relief. Self-medication with naproxen 220 milligram (mg) twice daily for a month did not help. Referral to an ophthalmologist did not reveal papilledema or glaucoma. His past medical history was significant for hypertension and gastroesophageal reflux. His medications included losartan, amlodipine, hydrochlorothiazide and pantoprazole.

On examination, his blood pressure (BP) was 140/82 mmHg, pulse of 55 beats per minute, weight 173 pounds with a BMI of 26.3, with a height of five foot and eight inches. Precordial examination revealed no cardiac murmur and carotid artery auscultation revealed no evidence of a carotid bruit. His gait station and cadence were normal. Cranial nerve examination was entirely normal. Funduscopic examination was entirely normal. He had no tongue weakness or dysarthria. Gag reflex was active symmetrically, shoulder shrug strong and symmetric with normal torsional action of the sternocleidomastoid bilaterally, with no atrophy. Power measurement of upper and lower extremities was normal as measured by the medical research council (MRC) grading and graded at 5/5 throughout. He was able to stand from the seated position with arms folded and was able to stand on his heels and toes with ease. His deep tendon reflexes were lively throughout except for trace ankle jerks bilaterally. Sensory examination was normal to large and small fiber modalities in the big toes, with an absent Romberg sign. In summary, his neurological examination was non-focal with no evidence of papilledema or lower brainstem involvement; all pertinent negative findings for a cough headache disorder patient.

**Figure 1:**
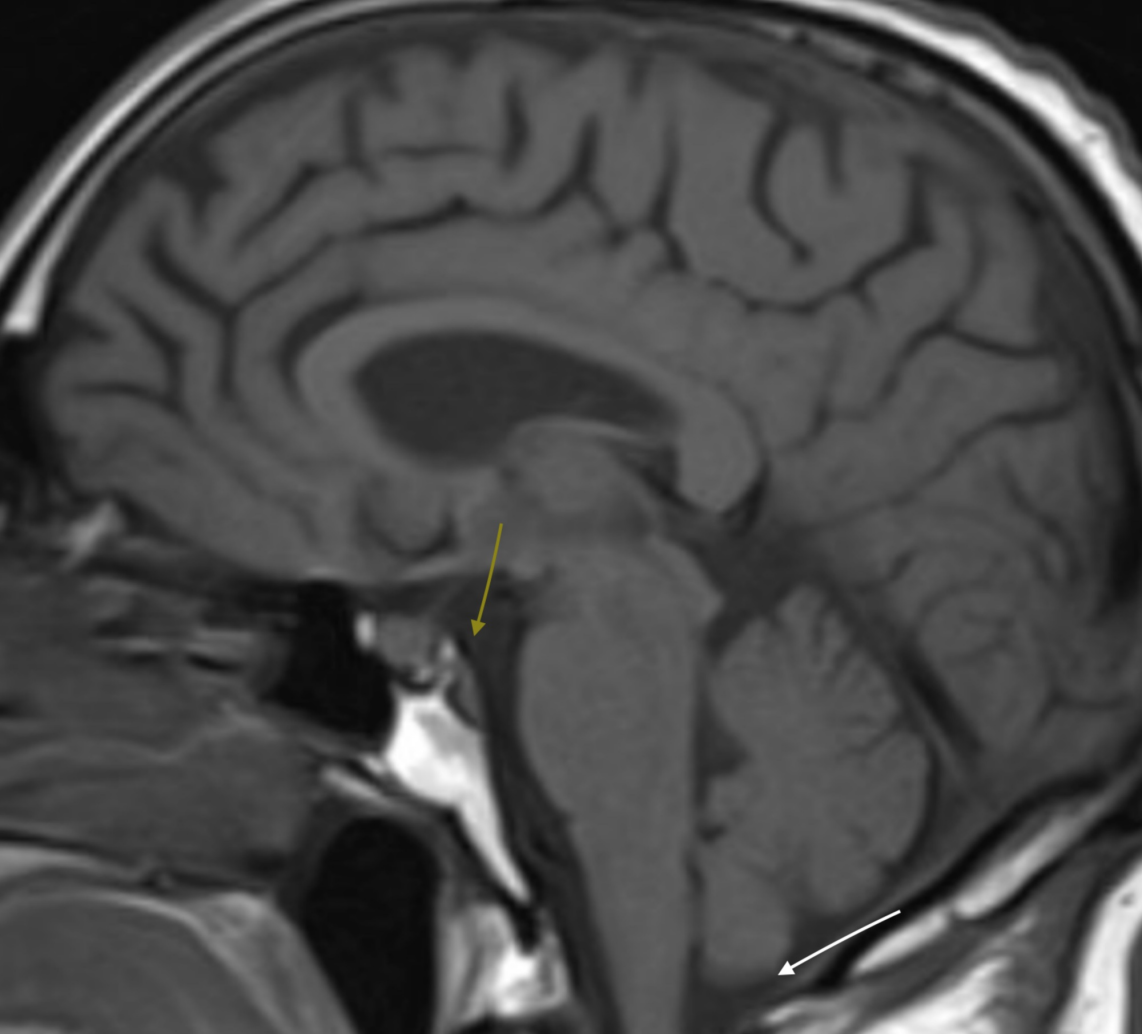
T1 sagittal MRI demonstrating capacious foramen magnum (white arrow) with generous basi-cranial flexion with absence of basilar impression (yellow arrow) MRI: magnetic resonance imaging

A magnetic resonance imaging (MRI) of the brain shows no evidence of a space-occupying lesion and there was no evidence of basilar impression or cerebellar ectopia on T1-weighted MRI sagittal section (Figure 1).

A complete blood count precluded any evidence of polycythemia or anemia. Serum chemistries and a thyroid screen were all normal. The patient underwent three therapeutic lumbar punctures (LP) over the course of 20 years; the patient was placed in the lateral recumbent position in the first two LPs and the sitting position for the last LP. The LPs were temporally spaced at twenty years, three years and three months. All three lumbar punctures registered a normal opening CSF pressure below 20 centimeter (cm) water, except the last lumbar puncture which was performed in the sitting position, but the CSF pressure at the lumbar level was below the CSF pressure registering at the foramen magnum. Three cc of CSF was drained with each LP, including the volume in the manometer. So on average, a total of three cc was drained with each LP. CSF chemistry for serum glucose, protein and cell count was tested for the first two lumbar punctures and was entirely normal. No LP headaches occurred. The resolution of cough headache lasted seventeen years with the first LP, three years with the second LP, and persistent headache resolution with the third LP until now (Table 1).

**Table 1:** Details of lumbar puncture procedures performed over the course of 20 years: centimeter (cm), cerebrospinal fluid (CSF)

YEAR PERFORMED	POSITION	OPENING PRESSURE	LENGTH OF HEADACHE RESOLUTION	VOLUME OF CSF DRAINED
2000	Lateral decubitus	15 cm water	17 years	3 cm
2017	Lateral decubitus	16 cm water	3 years	3 cm
2020	Sitting	26 cm water (lumbar), 31 cm water at foramen magnum	To be determined	3 cm

## Discussion

**Figure 2:**
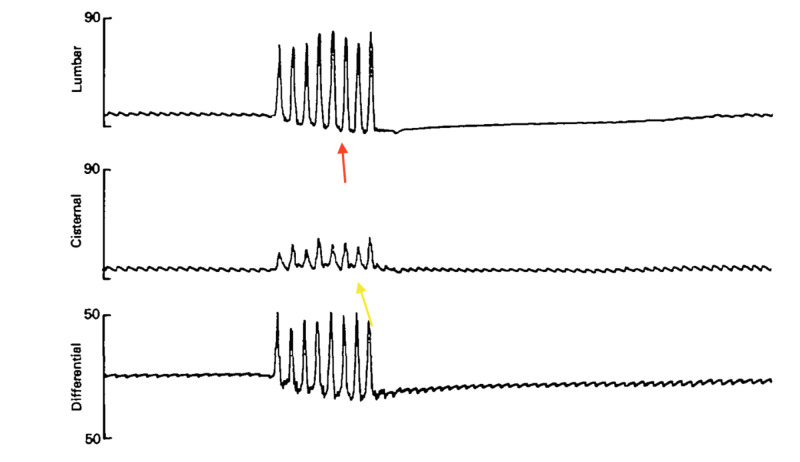
Cranio-spinal dissociation. Cisternal and lumbar pressure readings: pressure on the ordinate axis in millimeter mercury and time on abscissa in seconds. Drop of baseline pressure readings with eight consecutive coughs (red arrow) with lumbar readings but not so with cisterna magna readings (yellow arrow), where the baseline is flat

CSF pulsation arises from two sources: arterial pulsations and venous pulsations. The latter are modulated by changes in posture, respiration and Valsalva activities, such as coughing and straining. Venous pulsations are easily transferred into the CSF, as the venous membrane is softer and more pliable than the arterial wall. A pressure wave travels where there is a pressure differential. Two easily accessible nodes are the lumbar region and the cisterna magna; the lumbar pressure and cisterna magna pressure. Myelographic studies have suggested that following a cough, the pressure wave travels from the lumbar region upwards. One would expect that the traveling pressure wave dissipates energy as it moves upwards and the amplitude decreases in amplitude as it reaches the cisterna magna. Also the spine and head behave like a pipe organ, with the head containing compressible and distensible veins with high capacitance. This would lower cisternal pressure. Based upon lumbar and cisternal pressure readings performed in human subjects with cerebellar ectopia, it was shown that the cerebellar tonsils behave like a one-way valve, allowing the pressure wave to climb upwards through the foramen magnum, but that the rebound wave is blocked at the foramen magnum upon reflection in the cranium [5]. This would compound the jamming of the cerebellar tonsils in the foramen magnum, and this would increase the pressure differential between the lumbar spine and cisterna magna. This was proposed as a mechanism for cough headaches [6]. Relief of cough headaches was reported in patients who received pneumo-encephalograms, where spinal fluid is drained from the subarachnoid space and replaced with air [2]. This interference between the upward wave from a cough and the rebound downward wave from the cranium caused by a valve-like blockage at the foramen magnum is known as cranio-spinal pressure dissociation (Figure 2) [7].

**Figure 3:**
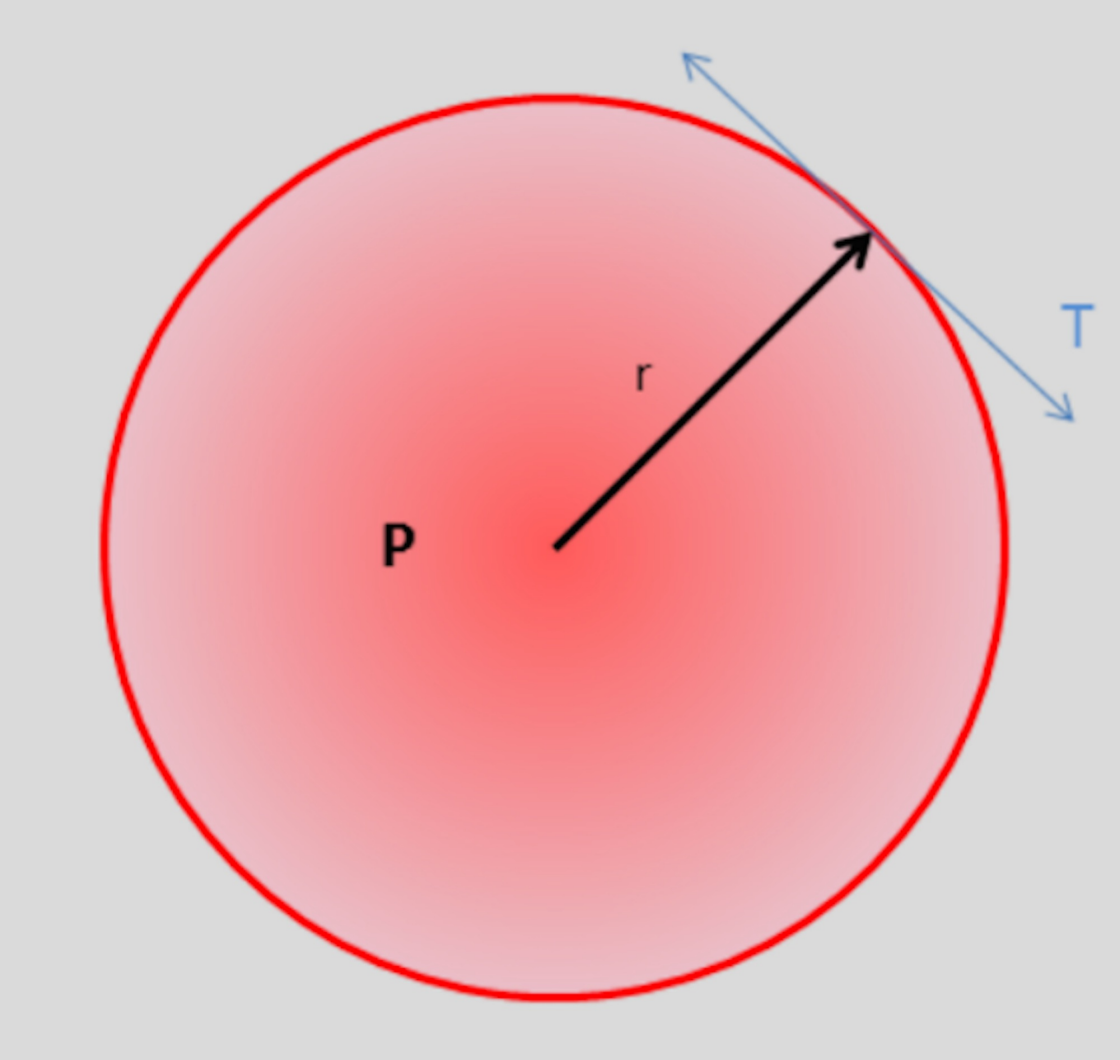
Law of Laplace. P = T.r: T is surface tension, P is the distention pressure and r is the radius of the sphere

So drainage of CSF fluid may lower the pressure differential between lumbar spine and the cisterna magna. Next, we will theoretically explain the reason why low volume CSF drainage may lower this pressure differential without resorting to high volume drainage. We will deploy the law of Laplace from physics and the algebraic formula for the volume of a sphere. In this hypothetical consideration, we will assume that the brain is a sphere of dura filled with fluid. The law of Laplace states that P = T.r, where T is the surface tension of the sphere, that is, the surface tension of the dura, P is the distention pressure across the dura, and r is the radius of the sphere, (Figure 3).

This sac of dura is encased by a bony case, a non-distensible skull. The same is true for the spinal canal which is enclosed by a bony cylinder. In the equilibrium state, the variations in the radius, r, of the dural sac are small indeed. Hence the small drift in the baseline of the lumbar spinal pressure as seen in Figure 3. By the Monroe-Kelley doctrine, the sum volumes brain, CSF and intracranial blood are normal. A drop in CSF volume is usually compensated by a rise in venous blood volume [8]. Hence, in our opinion, the direct effects of CSF drainage are but one factor that may lead to relief of cough headaches and may explain why low volume CSF drainage may be just as effective as high volume CSF drainage. There may be other dynamical factors. We believe that the hole created by the lumbar puncture may slightly lower the lumbar spinal pressure and reduce the magnitude of the upward wave and lower the lumbar-cisterna magna pressure differential, which then reduces the rebound wave and potentially reverses the spino-cranial dissociation. The valve mechanism at the foramen magnum is hence a cumulative effect overtime. This is speculation and needs to be borne out by direct lumbar and cisterna magna pressure readings. The reason why CSF drainage has a long-lasting effect is yet unknown. One can speculate that resetting the lumbar-cisterna magna pressure differential achieves a new equilibrium, which may be perturbed by a yet undetermined trigger that may lead to a cough headache. 

Indomethacin is well known to reduce intracranial pressure and reduce cerebral blood flow by inhibiting the formation of vasodilating prostaglandins via cyclo-oxygenase inhibition. This effect does not lead to ischemia as lactate is not produced and cerebral autoregulation is preserved. This effect is not demonstrated by other non-steroidal anti-inflammatory drugs [9]. However, indomethacin's wide range of side effects including hypertension, peptic ulcer disease and electrolytes abnormalities limits its usefulness. As noted by Williams [5], there is a phase during which lumbar pressure exceeds cisterna magna pressure, a net upward pressure wave, followed by one in which cisterna magna pressure exceeds lumbar pressure, a net downward pressure wave, the rebound wave. Williams and Wang propose that patients with PCHD may have CSF hypervolemia that, although not leading to increased ICP, may cause a higher pressure gradient in the rebound wave and a subsequent compression of the dura with coughing, the " jamming effect " at the foramen magnum [5,10]. Acetazolamide, by reducing CSF formation decreases the pressure differential and relieves the headache [10]. Indomethacin may also have a similar mechanism. However, a lumbar puncture, by reducing the upward pressure wave may reduce the rebound wave.

## Conclusions

PCHD is ipso facto a diagnosis of exclusion. The absence of papilledema and the absence of both basilar impression and a space-occupying lesion on MRI imaging of the brain are prerequisites. CSF lowering measures include pharmacotherapy with indomethacin or acetazolamide, which are the mainstay of therapy. However, CSF drainage with LP provides immediate and long-lasting relief. CSF dynamics are inherently complex due to non-linear mechanisms involving a matrix of forces; venous and arterial pressure; both intra- and extra-cranially, CSF pressure itself, autoregulatory mechanisms, chemoreceptors, baroreceptors, postural changes, effects of respiration etc. In this article, we attempt to reconcile the main ideas pertaining to the underlying mechanisms of benign cough headaches and propose that low volume CSF drainage may be as effective as high volume CSF drainage. This of course needs to be borne out by further studies. The concept of cranio-spinal dissociation is fleshed out in its full glory. Fortunately, this is a hypothesis that has been borne out by in-vivo measurements. The take-home message is that PCHD may resolve with a simple low volume drainage LP.
